# Integration of ATAC-seq and RNA-seq unveils transcription factors involved in low-nitrogen tolerance in cucumber

**DOI:** 10.3389/fpls.2026.1798277

**Published:** 2026-03-26

**Authors:** Bowen Li, Linhao Ma, Huaxiang Wu, Yike Han, Nan Liu, Ce Liu, Shengli Du, Aimin Wei

**Affiliations:** 1 College of Life Science, Nankai University, Tianjin, China; 2 Cucumber Research Institute, Tianjin Academy of Agricultural Sciences, Tianjin, China; 3 State Key Laboratory of Vegetable Biobreeding, Tianjin Academy of Agricultural Sciences, Tianjin, China

**Keywords:** ATAC-seq, CsTGA7, cucumber, low-nitrogen stress, RNA-Seq, transcriptional regulation

## Abstract

**Introduction:**

Nitrogen (N) availability is one of the major factors limiting cucumber (*Cucumis sativus L.*) growth and productivity. However, the hierarchical transcriptional and chromatin-based mechanisms underlying low-nitrogen (LN) adaptation remain unclear.

**Methods:**

This study investigated the dynamic molecular responses to LN stress and identified key regulatory factors by integrating time-course RNA-seq and ATAC-seq analyses in a tolerant accession (C52) and a sensitive accession (C13).

**Results:**

LN treatment induced distinct physiological and transcriptional responses: C52 activated a robust "resource-recycling" system, whereas C13 exhibited global chromatin tightening. We identified the bZIP transcription factor *CsTGA7* as a core regulator. Functional validation using VIGS demonstrated that *CsTGA7* is essential for LN tolerance, maintaining nitrate homeostasis and sustaining stress-responsive gene expression.

**Discussion:**

These findings provide new insights into the chromatin accessibility-mediated regulation of LN responses and identify *CsTGA7* as a promising target for breeding low-nitrogen-tolerant cultivars.

## 1 Introduction

Cucumber (*Cucumis sativus* L.) is one of the most globally important vegetable crops (Food and Agriculture Organization of the United Nations, 2024), yet its shallow root system makes its production heavily dependent on intensive nitrogen (N) fertilization (Wang et al., 2025a). While N inputs are essential for driving plant growth, photosynthesis, and protein synthesis, excessive application significantly increases production costs and triggers severe environmental issues, including nitrate leaching and soil eutrophication (Schulte-Uebbing et al., 2022; Gu et al., 2023; Wang et al., 2025b). Conversely, N deficiency leads to leaf chlorosis, growth inhibition, and the accumulation of reactive oxygen species (ROS), which can damage photosynthetic apparatus and limit yield potential (Mu and Chen, 2021a, MuChen, 2021b). Therefore, improving nitrogen use efficiency (NUE) and breeding cultivars with robust tolerance to low nitrogen (LN) stress are urgent priorities for sustainable horticulture (Krapp, 2015; Wani et al., 2021; Ciampitti et al., 2022).

carbon (C) and nitrogen metabolism are tightly coupled. Leaf chlorophyll content serves as a primary physiological indicator of N status, given that chlorophyll molecules act as substantial N reservoirs (Schlemmer et al., 2013; Feng et al., 2015). Moreover, the energy (ATP) and reducing power (NADPH) generated through light harvesting and electron transport are prerequisites for nitrate reductase activity and subsequent N assimilation (Sawhney et al., 1978; Nunes-Nesi et al., 2010; Yu et al., 2025). Disruption of this process under N starvation often triggers oxidative stress, necessitating robust antioxidant mechanisms to maintain cellular homeostasis (Huang et al., 2019; Sakuraba, 2022). Consequently, chlorophyll content and proxy metrics, such as SPAD values, are widely employed to phenotype LN tolerance (Lin et al., 2010; Xiong et al., 2015; Gholizadeh et al., 2017). Despite this tight physiological coupling between carbon and nitrogen metabolism, the transcriptional regulators that integrate carbon, nitrogen, and redox homeostasis under N−limiting conditions remain largely unexplored in cucumber.

In major staple crops and model species, significant progress has been made in deciphering N signaling networks. For instance, NODULE INCEPTION-like proteins (NLPs) have been identified as master regulators orchestrating root architecture and N uptake (Konishi and Yanagisawa, 2013; Liu et al., 2022), while the *NRT2/NAR2* modules mediate high-affinity nitrate transport (Cerezo et al., 2001; Remans et al., 2006; Kiba et al., 2012; Kotur et al., 2012; Varala et al., 2018). These findings highlight that improving NUE requires dissecting the hierarchical transcriptional network—from signal perception to metabolic adjustment (Lu et al., 2025). However, in contrast to model plants such as Arabidopsis and rice, the transcriptional—and, critically, the chromatin-based—mechanisms that govern dynamic nitrogen responses in cucumber remain largely unexplored.

Chromatin accessibility represents a pivotal layer of gene regulation that determines the access of transcription factors (TFs) to cis-regulatory elements, thereby gating gene expression (Bell et al., 2011; Chen et al., 2024; Ertl, 2024). While RNA-seq captures the output of gene expression, it often fails to identify the upstream regulatory events. Integrating Assay for Transposase-Accessible Chromatin with high-throughput sequencing (ATAC-seq) with RNA-seq overcomes this limitation by directly linking chromatin dynamics to transcriptional changes (Li et al., 2023, Li et al., 2025; Liu et al., 2023; Tian et al., 2024). In this study, to dissect the regulatory landscape governing N starvation, we selected two cucumber accessions, C13 and C52, which exhibit distinct physiological sensitivity to low nitrogen stress. While C52 maintains relative chlorophyll stability, C13 shows a more severe decline in SPAD values under N deficiency. Utilizing a multi-omics approach, we compared their early molecular responses using time-course RNA-seq and ATAC-seq. This work elucidates the chromatin accessibility-mediated molecular mechanisms of low nitrogen tolerance in cucumber and identifies a promising molecular target for improving NUE.

## 2 Materials and methods

### 2.1 Plant materials and growth conditions

Cucumber (*Cucumis sativus* L.) inbred lines, C13 and C52, were obtained from the Cucumber Research Institute of Tianjin Academy of Agricultural Sciences (PR China). A standardized hydroponic culture system was established to investigate the physiological responses and molecular mechanisms underlying nitrogen deficiency. Seeds were heat-treated at 65 °C for 15 min, followed by imbibition at room temperature for 2 h. Germination was carried out on moist filter paper at 28 °C in darkness for 2 days. Upon radicle emergence, uniform seeds were transferred to a water-based culture system at 25 °C until the cotyledons fully expanded. Subsequently, seedlings were transplanted into hydroponic containers and acclimated in half-strength Hoagland’s solution for 7 days, followed by full-strength solution until the one- or two-true-leaf stage. To investigate the specific effects of nitrogen deficiency, two distinct nutrient treatments were established with precise ion balancing: Normal Nitrogen (NN): A standard Hoagland solution with a total nitrogen concentration of 15 mM. The major macro-salts included 945 mg/L Ca(NO_3_)_2_·4H_2_O, 607 mg/L KNO_3_, 115 mg/L NH_4_H_2_PO_4_, and 493 mg/L MgSO_4_·7H_2_O. Low Nitrogen (LN): A modified nutrient solution with a total nitrogen concentration reduced to 1 mM (supplied by adding 0.5 mM NH_4_NO_3_ to a nitrogen-free base). To ensure that the phenotypic differences were solely attributable to nitrogen availability, other essential macro-elements were maintained at equivalent levels to the NN treatment by substituting nitrogen-containing salts with: 350 mg/L CaCl_2_, 200 mg/L KCl, 350 mg/L KH_2_PO_4_, and 493 mg/L MgSO_4_·7H_2_O. Both treatments were supplemented with identical micronutrients (Solution C), consisting of 2.86 mg/L H_3_BO_3_, 1.61 mg/L MnSO_4_·H_2_O, 0.22 mg/L ZnSO_4_·7H_2_O, 0.03 mg/L Na_2_MoO_4_·2H_2_O, and 36 mg/L Fe-Na-EDTA. The pH of the nutrient solutions was adjusted to 5.8 ± 0.1 using 0.1 M KOH or HCl. Solutions were renewed every 3 days to maintain nutrient consistency and adequate aeration.

### 2.2 Physio-biochemical assays

Relative chlorophyll content was determined non-destructively using a portable chlorophyll meter (SPAD-502Plus, Konica Minolta, Japan). To minimize environmental variation, all measurements were conducted under consistent light and temperature conditions. For each leaf, SPAD values were recorded from five representative positions (leaf tip, base, margins, and center, avoiding major veins), and the average was calculated to represent the final SPAD value of the leaf. Fresh leaf tissues were ground into fine homogenate in liquid nitrogen for biochemical analysis. To assess nitrogen accumulation, nitrate (NO_3_^-^) and ammonium (NH_4_^+^) contents were quantified using colorimetric methods based on the salicylic acid reaction. The extraction and determination were performed using the Plant Nitrate Nitrogen Assay Kit (NMQ0710) and Plant Ammonium Nitrogen Assay Kit (NMW0748) (Nuominkeda, China), respectively, following the manufacturer’s instructions. Hydrogen peroxide (H_2_O_2_) accumulation was measured using a commercial H_2_O_2_ assay kit (BC3595, Solarbio, Beijing, China) according to the standard protocol.

### 2.3 RNA extraction and library preparation

RNA-seq libraries were constructed using the second expanding leaves of uniform C13 and C52 seedlings. The experiment included two time points (4 h and 30 h) under two nitrogen regimes, designated as follows: (1) 4H_NN: 4 h in normal Hoagland’s solution (Control); (2) 4H_LN: 4 h in nitrogen-free Hoagland’s solution (Low Nitrogen); (3) 30H_NN: 30 h in normal Hoagland’s solution (Control); (4) 30H_LN: 30 h in nitrogen-free Hoagland’s solution (Low Nitrogen). Three independent biological replicates were performed for each treatment resulting in a total of 24 independent RNA-seq libraries to ensure high statistical power and reproducibility. Total RNA was extracted using the RNAprep Pure Plant Plus Kit (Polysaccharides & Polyphenols-rich) (DP441, TIANGEN, Beijing, China). RNA integrity and purity were assessed using the Agilent 5400 Fragment Analyzer system (Agilent Technologies, USA). Sequencing libraries were constructed using the Fast RNA-seq Lib Prep Kit V2 (RK20306, ABclonal, Wuhan, China) with a specific modification to the standard protocol. The VAHTS mRNA Capture Beads (N401-01/02, Vazyme, Nanjing, China) were used for poly(A) mRNA enrichment and fragmentation, substituting for the reagents specified in section. RNA Enrichment and Fragmentation” of the ABclonal kit. All other steps were performed strictly according to the manufacturers’ instructions.

### 2.4 RNA sequencing and data analysis

Raw image data obtained from high-throughput sequencing were converted into raw reads (FASTQ format) via CASAVA base calling. To ensure data consistency and minimize analysis bias across sequencing batches, all raw data were processed using a unified pipeline by Novogene. Strict quality control was performed to filter the raw reads. Reads containing adapters, reads with undetermined bases (N), and low-quality reads (where bases with Qphred ≤ 5 accounted for > 50% of the read length) were removed to generate clean reads. Reference genome indexing and read alignment were performed using HISAT2 (v2.0.5) (Kim et al., 2015) against the Cucumis sativus reference genome (Chinese Long v3) (Yu et al., 2023). The mapped read counts for each gene were calculated using featureCounts (v1.5.0-p3) (Liao et al., 2014). Gene expression levels were subsequently normalized to FPKM (Fragments Per Kilobase of transcript per Million mapped reads) based on gene length. Differential expression analysis between treatment groups was performed using DESeq2 (v1.20.0) (Love et al., 2014). PCA was executed using the prcomp() function and visualized with the ggplot2 R package to examine the clustering patterns among genotypes and treatments. Pearson correlation coefficients (R) between all pairs of samples were calculated using the cor() function in R with the parameter method = “pearson”. Differentially Expressed Genes (DEGs) were identified based on the criteria of |log_2_ fold change| > 1 and *P*-value < 0.05. Functional annotation and enrichment analysis of DEGs were conducted using the Gene Ontology (GO) (Ashburner et al., 2000; Gene Ontology Consortium, 2026) and Kyoto Encyclopedia of Genes and Genomes (KEGG) databases (Kanehisa et al., 2025). Weighted Gene Co-expression Network Analysis (WGCNA) was performed using the WGCNA R package to identify co-expressed gene modules (Langfelder and Horvath, 2008). To ensure statistical robustness, all 24 independent transcriptome profiles (2 accessions * 2 treatments * 2 time points * 3 replicates) were used as input. To identify modules associated with nitrogen tolerance, phenotypic data were converted into a numeric matrix, with the tolerant accession C52 assigned a value of 1 and the sensitive accession C13 assigned a value of 0. A signed adjacency matrix was constructed using an optimized soft-thresholding power. Additionally, Protein-Protein Interaction (PPI) networks were constructed based on the STRING database and visualized using Cytoscape (Shannon et al., 2003; Szklarczyk et al., 2021).

### 2.5 ATAC-seq library preparation

ATAC-seq libraries were prepared from the second expanding leaves of cucumber seedlings at the two-leaf stage, harvested under both control and low nitrogen conditions (4 h treatment). Two biological replicates were performed for each condition. Nuclei isolation was performed following previously described protocols with minor modifications (Buenrostro et al., 2013; Corces et al., 2017; Bajic et al., 2018). Briefly, fresh leaf tissues were chopped in 1 mL of 1×Homogenization Buffer (HB), filtered, and centrifuged at 500×g for 5 min at 4 °C. The pellet was resuspended and subjected to density gradient centrifugation for 10 min to remove cellular debris. The purified nuclei suspension was quantified using a LUNA-FL automated cell counter. Approximately 50, 000 nuclei were aliquoted, washed with 1 mL of wash buffer, and centrifuged at 500×g for 5 min. The resulting nuclei pellet was resuspended in the Tn5 transposase reaction mixture. The transposition reaction was incubated at 37 °C for 30 min to tag and fragment accessible chromatin. Following transposition, equimolar amounts of Adapter 1 and Adapter 2 were added, and the library was amplified by PCR using the ATAC-seq Novogene Kit (Novogene, China). The PCR products were purified using AMPure XP beads, and library quality was assessed using a Qubit fluorometer. The final libraries were sequenced on the Illumina NovaSeq platform by Novogene Bioinformatics Technology Co., Ltd. (Beijing, China) to generate 150-bp paired-end reads.

### 2.6 ATAC-seq data processing

Raw sequencing reads were pre-processed using fastp (v0.20.0) to remove adapters and low-quality bases (Chen et al., 2018). The resulting clean reads were aligned to Cucumber (Chinese Long) v3 reference genome using BWA (v0.7.12) (Li and Durbin, 2009). To reduce background noise, reads mapping to mitochondrial and chloroplast DNA were excluded. The alignment files were further filtered to retain high-quality, uniquely mapped reads (MAPQ≥13) that were properly paired. PCR duplicates were removed to ensure data accuracy. Peaks were identified using MACS2 (v2.1.0) with parameters specifically adjusted for ATAC-seq fragment characteristics: -q 0.05 --call-summits -Nomodel --shift -100 --extsize 200 --keep-dup all (Zhang et al., 2008). To allow for comparison across samples, peaks from all groups were merged using bedtools merge to generate a consensus peak set (Quinlan and Hall, 2010). The read counts for each peak were calculated using bedtools coverage and normalized to Reads Per Million mapped reads (RPM). The Pearson correlation between biological replicates was calculated using deepTools (v3.0.2) (Ramírez et al., 2016). For differential analysis, the mean RPM of biological replicates was calculated for each group. The log_2_ fold change (Log_2_FC) was computed as log_2_(RPM_treatment_/RPM_control_). DARs were defined based on the criteria of |log_2_(fold change)| ≥ 0.5 and *P* < 0.05. Genomic annotation of the identified peaks was performed using the ChIPseeker (Yu et al., 2015).

### 2.7 Motif analysis and regulatory network construction

To provide sufficient genomic context and incorporate flanking regulatory elements, sequences spanning 1000 bp (centered on the summits of C52-specific DARs, ± 500 bp) were extracted. Motif enrichment was performed using the findMotifsGenome.pl program in HOMER (v4.9.1) (Heinz et al., 2010). To ensure precise detection across variable motif lengths and normalization, the analysis was executed with the following specific command-line parameters: -len 8, 10, 12, 14 -gc -size given -p 2 -S 25 -homer2 -dumpFasta. Motifs with a q-value < 0.05 were considered significant and selected for subsequent analysis. Complementary motif discovery was performed using the MEME-ChIP suite (Machanick and Bailey, 2011). We queried the JASPAR CORE (Plants and Arabidopsis) (Fornes et al., 2020) and CIS-BP 2.00 (Single Species: Cucumis sativus) databases (Weirauch et al., 2014). For each submission, MEME was configured to identify a maximum of 15 motifs using the ZOOPS (Zero or One Occurrence Per Sequence) model. The motif width was constrained between 6 and 15 bp, and the search size was set to 100, 000 bp. Motifs were filtered based on an E-value < 0.05 and a CentriMo score > 5.0. Subsequently, FIMO was utilized to scan the genomic sequences for specific binding sites, retaining only occurrences with a q-value < 0.05 (Gupta et al., 2007). We utilized two specific database groupings to maximize annotation coverage: (1) the Cucumis sativus specific database (CIS-BP 2.00), and (2) the Arabidopsis thaliana and Core Plants databases (Fornes et al., 2020). Significant matches were filtered based on an E-value threshold of < 0.05. Subsequently, the Position Weight Matrices (PWMs) of the identified TFs were used to scan the background sequences using FIMO to locate specific binding sites (Grant et al., 2011). Only motif occurrences with a q-value < 0.05 were retained to establish TF-DAR interactions.To cross-validate the regulatory relationships, we performed an independent binding site prediction using PlantRegMap with the species parameter set to Cucumis sativus (Tian et al., 2020). Predicted TF-target pairs with a q-value < 0.05 were selected. Finally, a Venn diagram was generated to visualize the overlap of TF-target relationships predicted by HOMER, MEME Suite, and PlantRegMap. Only high-confidence interactions supported by the intersection of these methods were used to construct the transcriptional regulatory network (TRN), which was visualized using Cytoscape.

### 2.8 Virus-induced gene silencing assays

The TRSV (Tobacco Ringspot Virus)-based VIGS system was employed for functional verification (Fang et al., 2021). A 300-bp gene-specific fragment was designed using the SGN VIGS Tool (Fernandez-Pozo et al., 2015) and cloned into the TRSV2 vector via homologous recombination to generate the TRSV2-Target construct. For inoculation, the TRSV2-Target construct was co-inoculated with the auxiliary vector TRSV1 into cucumber seedlings via Agrobacterium infiltration as described previously (Wu et al., 2025). Two control groups were established: plants co-inoculated with the empty TRSV2 vector (TRSV2-EV) and TRSV1 served as the negative control, while those inoculated with TRSV2 carrying the phytoene desaturase gene (TRSV2-PDS) served as the phenotypic marker (positive control). EfficiencyTwo to three weeks post-inoculation (wpi), the success of the VIGS system was monitored by the appearance of a photobleaching phenotype in the true leaves of TRSV2-PDS plants. Subsequently, leaf samples were collected from TRSV2-Target and TRSV2-EV plants for RNA extraction and qRT-PCR analysis. To ensure the reliability of phenotypic analysis, silencing efficiency was quantified. Plants exhibiting a transcript reduction of ≥50% relative to the median expression level of the TRSV2-EV control were identified as effectively silenced lines and selected for further phenotypic characterization. The sequences of all primers used for VIGS cloning are listed in **Supplementary Table 1**.

### 2.9 Sequence analysis and phylogenetic characterization

To characterize the evolutionary relationship and conserved domains of CsTGA7, comprehensive bioinformatic analyses were performed. Amino acid sequences of TGA7 transcription factors from Cucumis sativus and Arabidopsis thaliana were retrieved from the Cucurbit Genomics Database (CuGenDB) and TAIR, respectively. Sequence Alignment and Domain Prediction: Multiple sequence alignment was conducted using Clustal Omega (Sievers et al., 2011) to identify conserved residues. Pairwise sequence identity and similarity between cucumber and Arabidopsis TGA7 homologs were calculated using the EMBOSS Needle tool (Rice et al., 2000) based on the BLOSUM62 scoring matrix. The conserved protein domain architecture was predicted using the SMART database (Letunic et al., 2021). A Maximum Likelihood (ML) phylogenetic tree was constructed using MEGA 11 (Tamura et al., 2021) with 1, 000 bootstrap replicates to ensure node reliability.

### 2.10 qRT-PCR validation

Total RNA was isolated from plant tissues using the Magen RNA Extraction Kit (Magen, China) following the manufacturer’s instructions. First-strand cDNA was synthesized using the PerfectStart Uni RT & qPCR Kit (TransGen Biotech, Beijing, China). Quantitative real-time PCR (qRT-PCR) was performed to evaluate gene expression levels. EF1A and F-BOX were selected as internal reference genes for data normalization. The relative expression levels were calculated using the 2 ^-△△Ct^ method, with the TRSV-EV plants under normal nitrogen conditions (NN) serving as the calibrator. The sequences of all primers used for qRT-PCR are listed in **Supplementary Table 1**.

## 3 Results

### 3.1 Distinct phenotypic divergence between C13 and C52 under low-nitrogen stress

**Figure 1:**
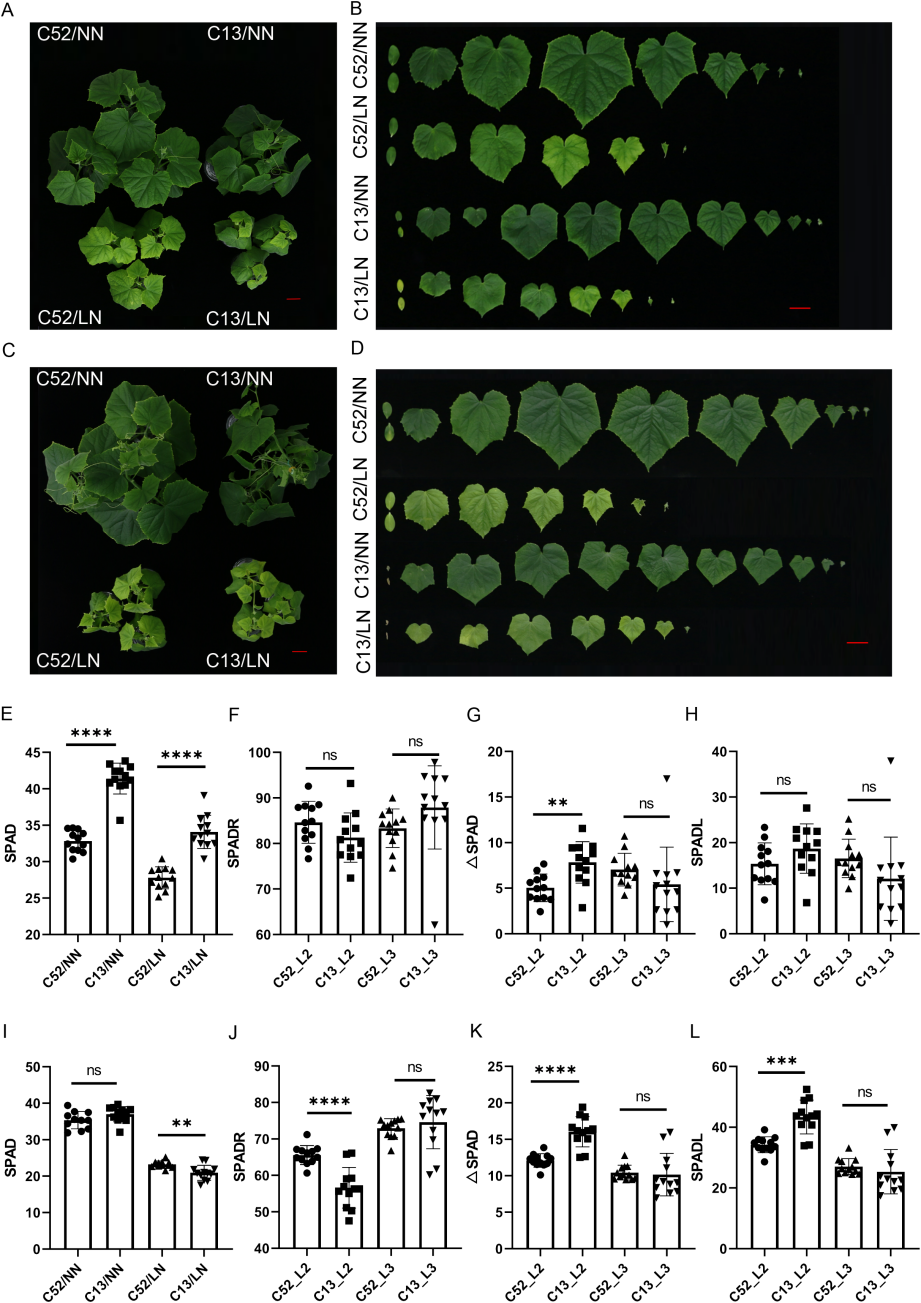
Phenotypic and physiological characterization of cucumber accessions C52 (tolerant) and C13 (sensitive) under low nitrogen stress. **(A, B)** Phenotypes of seedlings grown under normal nitrogen (NN) and low nitrogen (LN) conditions for 10 days. **(C, D)** Phenotypes of seedlings at 15 days after treatment (DAT). **(E)** SPAD values of the second newly emerged leaf (L2) at 10 DAT. **(F–H)** Comparative analysis of derived chlorophyll indices in the second (L2) and third (L3) leaves at 10 DAT, including relative SPAD ratio (SPADR) **(F)**, SPAD difference (△SPAD) **(G)**, and relative SPAD difference (SPADL) **(H, I)** SPAD values of the L2 leaf at 15 DAT. **(J–L)** Evaluation of derived chlorophyll indices in L2 and L3 leaves at 15 DAT, including SPADR **(J)**, △SPAD **(K)**, and SPADL **(L)**., highlighting the significant divergence in tolerance between the two accessions. Data are presented as mean ± SD (n = 10). Significant differences are indicated by asterisks (**P < 0.01; ***P < 0.001; ****P < 0.0001). 'ns' indicates no significant difference.

To evaluate the low nitrogen (LN) tolerance of the two cucumber accessions, C13 and C52 seedlings were subjected to LN and normal nitrogen (NN) conditions for a duration of 10 days (**Figures 1A, B**) and 15 days (**Figures 1C, D**). Phenotypic divergence became increasingly apparent as the stress persisted. To quantify leaf chlorosis—a characteristic symptom of N deficiency—we monitored the chlorophyll content (SPAD values) of the second and third leaves from the shoot apex (representing the newly emerged leaves, designated as L2 and L3) at 10 and 15 days after treatment (DAT).

To comprehensively assess stress sensitivity, three derived indices—△SPAD (difference), SPADR (relative ratio), and SPADL (relative difference ratio)—were calculated. Higher △SPAD and SPADL values indicate severe chlorosis (lower tolerance), while a higher SPADR signifies robust chlorophyll retention (higher tolerance). At 10 DAT, the physiological responses of the two accessions began to diverge (**Figures 1E–H**). Specifically, in L2 (the second leaf from the apex), C13 exhibited a significantly higher △SPAD compared to C52, indicating an earlier onset of chlorosis. However, no significant differences were observed in SPADL for L2, nor in any indices for the older L3 between the two accessions at this stage. This suggests that the younger, rapidly expanding L2 is more sensitive to early N deprivation than the relatively mature L3.

By 15 DAT, the disparity in LN tolerance became pronounced (**Figures 1I–L**). In L2, C13 displayed significantly higher △SPAD and SPADL values, coupled with a significantly lower SPADR compared to C52 (*P* < 0.05). Conversely, the indices for L3 showed no statistically significant differences between the varieties. These results demonstrate that C52 possesses a superior capacity to maintain chlorophyll homeostasis under N-limiting conditions, whereas C13 is highly sensitive to N deficiency. Furthermore, our data identify the second leaf from the shoot apex as the most effective phenotypic indicator for evaluating LN tolerance in cucumber seedlings. Based on these physiological distinctions, C52 (tolerant) and C13 (sensitive) were selected for subsequent multi-omics analyses.

### 3.2 Early transcriptional responses and WGCNA module analysis of C13 and C52 Under low-nitrogen stress

**Figure 2:**
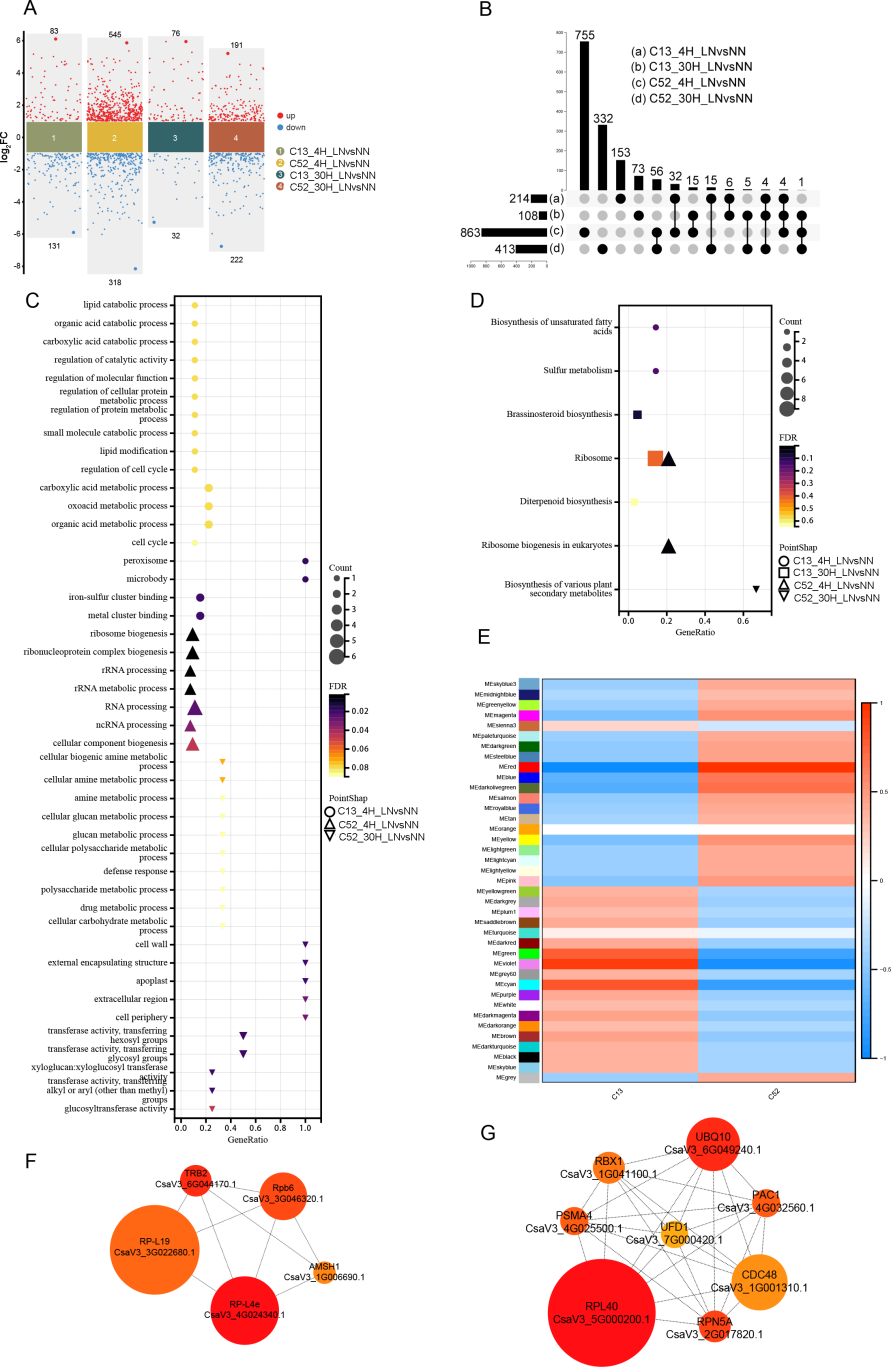
Comparative transcriptomic landscape and gene co-expression network analysis of C13 and C52 under low nitrogen stress. **(A)** Volcano plots illustrating the distribution of Differentially Expressed Genes (DEGs) in C13 and C52 leaves following 4 h and 30 h of low nitrogen (LN) treatment compared with normal nitrogen (NN). The x-axis represents the log_2_ fold change (log_2_FC), with positive and negative values indicating upregulation and downregulation, respectively. The selection criteria were *P* < 0.05 and |log_2_FC| > 1. **(B)** UpSet plot visualizing the intersection and uniqueness of DEGs across different comparisons, highlighting the divergent temporal response patterns between the two accessions. **(C, D)** Functional enrichment analysis of DEGs. Bubble plots representing the top enriched Gene Ontology (GO) terms **(C)** and KEGG pathways **(D)**. The size of the bubbles represents the gene count, and the color scale indicates statistical significance. **(E)** Module–trait relationship analysis derived from Weighted Gene Co-expression Network Analysis (WGCNA). The phenotypic trait was defined based on N-tolerance (C52 = 1, C13 = 0) to identify modules specifically associated with the tolerant or sensitive genotype. **(F, G)** Protein–Protein Interaction (PPI) networks of hub genes within key modules. **(F)** The VIOLET module, highly correlated with the sensitive accession C13. **(G)** The RED module, highly correlated with the tolerant accession C52. Node size represents the degree of connectivity.

To investigate the molecular mechanisms underlying the divergent leaf SPAD phenotypes of C13 and C52 under nitrogen (N)-deficient conditions, we performed RNA-seq analysis on leaf tissues following 4 h and 30 h of LN or NN treatment. Differentially expressed genes (DEGs) were identified using a threshold of *P*-value < 0.05 and |log_2_FC| > 1. As shown in **Figures 2A**, early transcriptional reprogramming was evident in both accessions. At 4 h, C13 exhibited 83 upregulated and 131 downregulated genes, whereas C52 displayed a much broader response with 545 upregulated and 318 downregulated genes. By 30 h, the number of DEGs in C13 decreased to 76 upregulated and 32 downregulated genes, while C52 maintained a robust response with 191 upregulated and 222 downregulated genes. These results indicate that N-adaptive regulatory networks are activated during the initial phase of N deprivation.

An UpSet plot analysis (**Figure 2B**) revealed distinct patterns in gene regulation stability. C52 maintained a substantial number of unique DEGs at both 4 h and 30 h, suggesting the formation of a stable and persistent regulatory network to cope with N deficiency. In contrast, C13 exhibited consistently fewer DEGs at both time points, implying the absence of an effective N-responsive regulatory mechanism or a failure to fully activate relevant pathways. This transcriptional inertia likely compromises the adaptive capacity of C13, correlating directly with the significant decline in SPAD values observed under LN stress.

Gene Ontology (GO) enrichment analysis (**Figure 2C**) revealed distinct metabolic strategies between the two accessions. At the early stage (4 h), DEGs in C13 were primarily enriched in catabolic processes, such as lipid and organic acid catabolism. This suggests C13 adopts an “energy mobilization” strategy, breaking down storage compounds to generate carbon skeletons and energy for basic survival. Conversely, C52 prioritized the remodeling of protein synthesis machinery, with DEGs significantly enriched in ribosome biogenesis and RNA processing. Although less statistically significant than the catabolic response in C13, this indicates a proactive adjustment of the translational landscape in C52. KEGG pathway analysis (**Figure 2D**) corroborated these findings, showing that the ribosome pathway was central to the C52 response at both 4 h and 30 h. Meanwhile, C13 initially focused on basal metabolism (e.g., unsaturated fatty acid biosynthesis, sulfur metabolism) and only shifted toward hormone signaling (brassinosteroids) and secondary metabolism (diterpenoids) by 30 h. Collectively, these results suggest that under LN conditions, C52 prioritizes the stability of protein synthesis to sustain key metabolic functions, whereas C13 resorts to catabolic breakdown to survive the “energy deficit, “ attempting to repair cellular structures via membrane-related metabolism.

To further resolve the regulatory architecture underlying these divergent responses, we performed Weighted Gene Co-expression Network Analysis (WGCNA). A module-trait relationship analysis was conducted to identify co-expression modules significantly correlated with the N-tolerance phenotypes of the two accessions (**Figure 2E**). This identified the RED module as highly correlated with the tolerant genotype C52, and the VIOLET module as highly correlated with the sensitive genotype C13. Protein-protein interaction (PPI) networks were constructed for genes within these modules (**Figures 2F, G**). In the C52-associated RED module (**Figure 2G**), the network is explicitly anchored by core components of the ubiquitin-proteasome system, including *PSMA4* (20S proteasome alpha subunit), *CDC48* (cell division cycle protein 48), *UBQ10* (polyubiquitin 10), and *RBX1* (RING-box protein 1). The centralization of these hubs provides molecular-level evidence that C52 actively mobilizes the proteasomal machinery to recycle intracellular nutrients under N stress. Conversely, the VIOLET module (C13-associated, **Figure 2F**) is defined by hubs such as *Rpb6* (RNA polymerase subunit 6) and *RPL19* (large subunit ribosomal protein L19). Although this module is globally enriched in chromatin assembly terms (as shown in GO analysis), the prominence of basal transcriptional and translational components (*Rpb6*, *RPL19*) as hubs—within a background of chromatin tightening—reinforces the hypothesis of a “stalled” or uncoordinated gene expression state in the sensitive accession.

**Figure 3:**
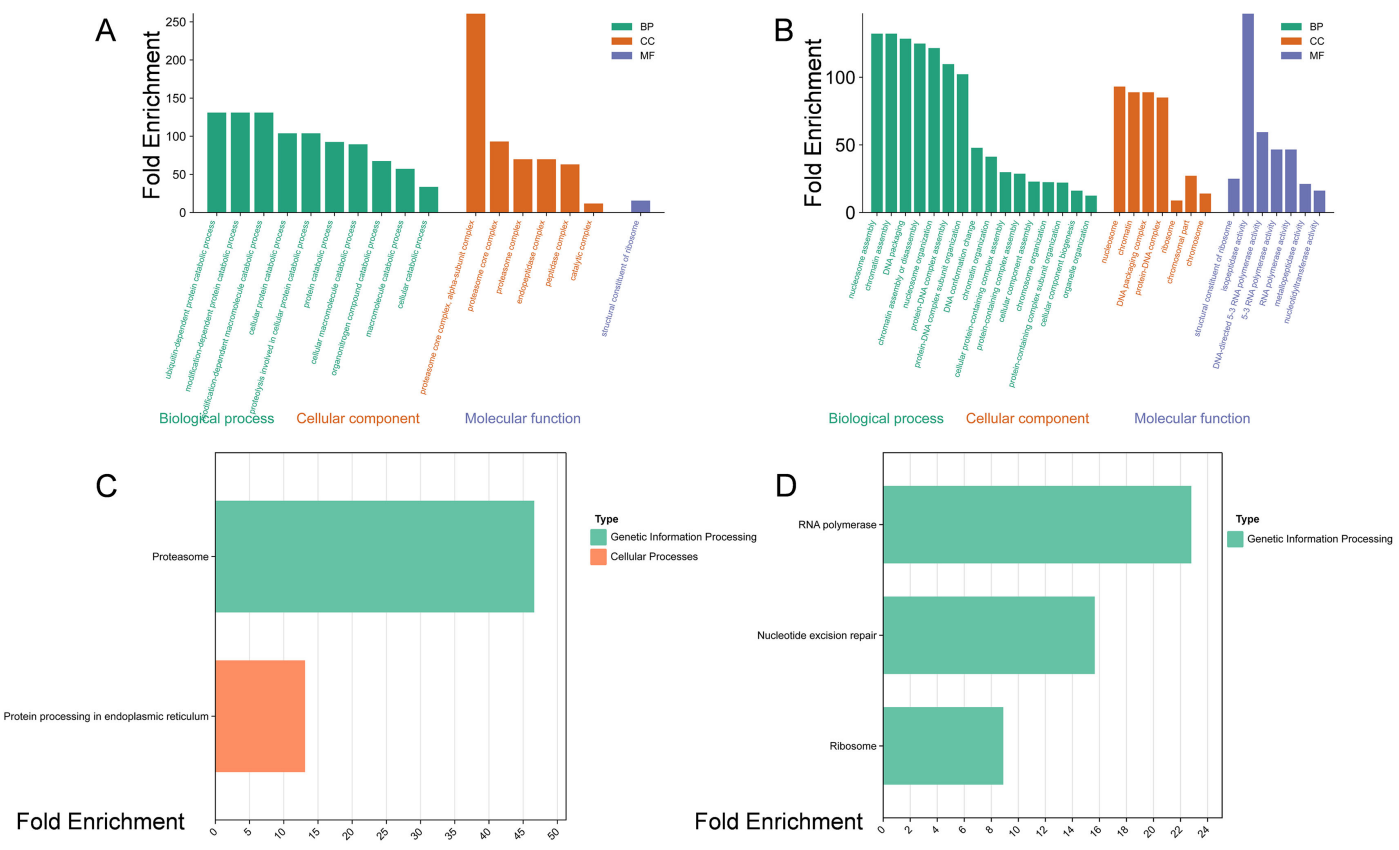
Functional characterization of distinct regulatory modules associated with N-tolerance (RED) and N-sensitivity (VIOLET). **(A)** Top significantly enriched Gene Ontology (GO) terms of the RED module (positively correlated with C52). **(B)** Top significantly enriched GO terms of the VIOLET module (positively correlated with C13). **(C)** Top significantly enriched KEGG pathways of the RED module. Note the prominent enrichment of terms related to the ubiquitin-proteasome system (UPS) and protein processing in the endoplasmic reticulum. **(D)** Top significantly enriched KEGG pathways of the VIOLET module. These terms are primarily centered on chromatin dynamics (e.g., nucleosome assembly, DNA packaging), suggesting a transcriptional repression or “dormant” state under stress. The *x-axis* represents the enrichment significance (e.g., -log10 P-value) or gene ratio, and the *y-axis* lists the specific functional categories.

Functional enrichment analysis of the RED module (**Figures 3A, C**) revealed a significant enrichment in the ubiquitin-proteasome system (UPS), characterized by terms such as “ubiquitin-dependent protein catabolic process, “ “proteasome core complex, “ and “proteasome.” This indicates that the N-tolerant C52 employs a sophisticated resource reallocation strategy rather than simple degradation. By upregulating the proteasome machinery, C52 likely degrades non-essential or damaged proteins to recycle amino acids, thereby fueling the synthesis of stress-responsive proteins and enhancing Nitrogen Use Efficiency (NUE). Additionally, the enrichment of “protein processing in endoplasmic reticulum” suggests that C52 maintains proteostasis by efficiently clearing toxic protein aggregates. Thus, the LN tolerance of C52 is underpinned by an active “resource recycling system” that facilitates efficient metabolic turnover.In contrast, the VIOLET module (**Figures 3B, D**), associated with C13, was enriched in chromatin dynamics, including “nucleosome assembly, “ “chromatin assembly, “ “DNA packaging, “ and “DNA conformation change.” The upregulation of these processes is typically indicative of heterochromatinization and transcriptional silencing. This suggests that C13 cells may enter a “dormant” or quiescent state, conserving energy by globally suppressing transcription, which inevitably retards growth. While chromatin compaction might offer passive protection against LN-induced DNA damage, it represents a defensive rather than adaptive strategy. Notably, although ribosome-related genes were also present in this module, the concurrent enrichment of chromatin assembly suggests uncoordinated regulation in C13: the potential demand for protein synthesis is restricted by chromatin tightening, leading to a failure in biomass accumulation.

In summary, C52 adapts to LN stress by activating protein turnover and recycling mechanisms to maintain cellular homeostasis and N supply. Conversely, C13 adopts a passive strategy characterized by chromatin remodeling and transcriptional repression, limiting its ability to mobilize resources rapidly and resulting in compromised LN tolerance.

### 3.3 Integration of ATAC-seq and RNA-seq unveils core transcription factors driving low-nitrogen adaptation

**Figure 4:**
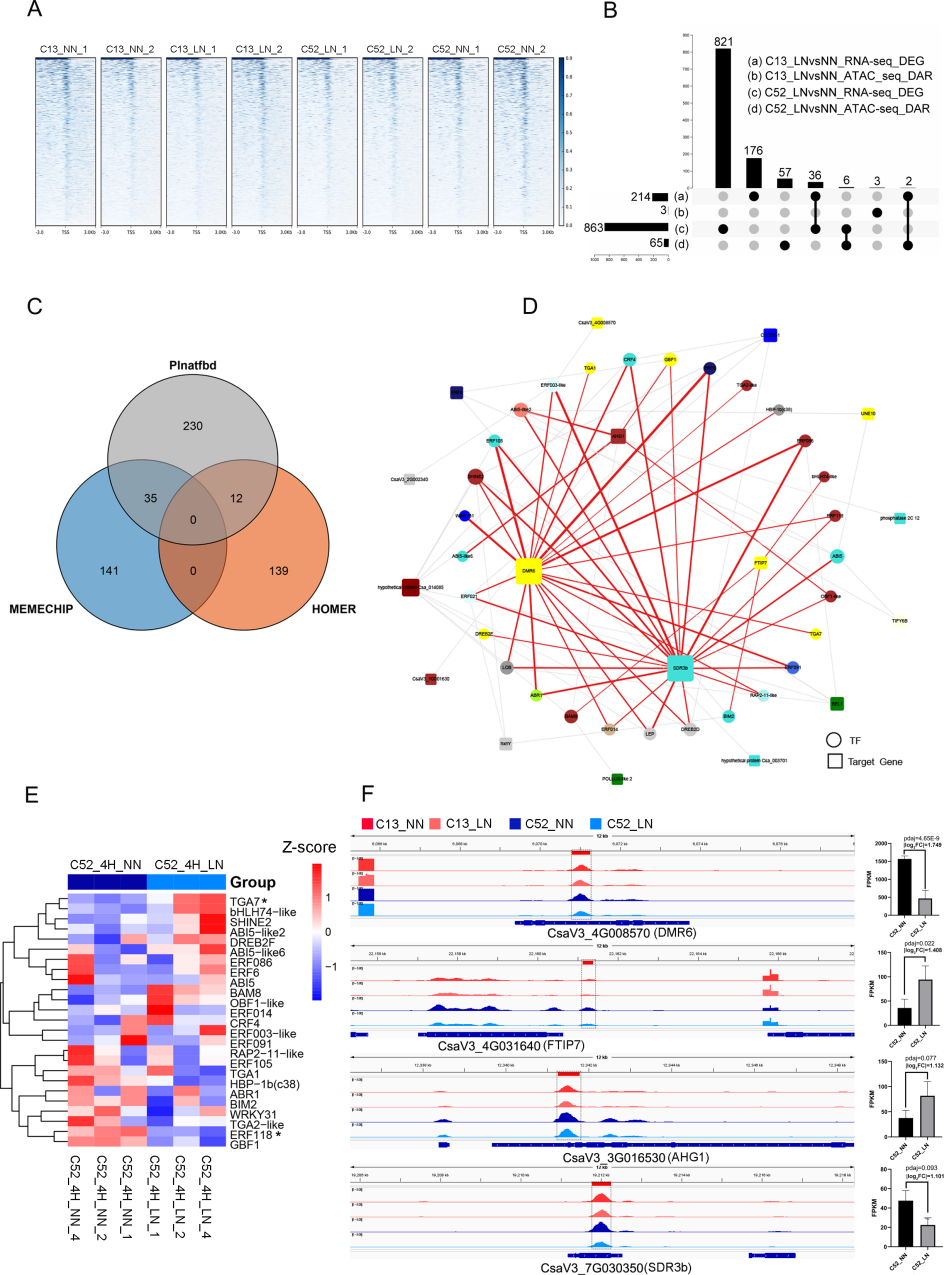
Integration of ATAC-seq and RNA-seq to decipher the core transcriptional regulatory network driving early low-nitrogen adaptation in C52. **(A)** Heatmap illustrating the genome-wide enrichment of ATAC-seq signals around Transcription Start Sites (TSS) in C13 and C52 leaves under normal (NN) and low nitrogen (LN, 4 h) conditions. The distinct enrichment patterns confirm high library quality and the effective capture of open chromatin regions. **(B)** UpSet plot visualizing the intersection between Differentially Accessible Regions (DARs) and Differentially Expressed Genes (DEGs) across different comparisons. The partial overlap indicates a complex, multilayered relationship between chromatin remodeling and transcriptional output. **(C)** Venn diagram showing the consensus of transcription factor (TF) binding motifs predicted by three complementary algorithms (HOMER, MEME-ChIP, and PlantTFDB) within the flanking sequences (± 1 kb) of DARs in C52. **(D)** The inferred Transcriptional Regulatory Network (TRN) for the early N-starvation response in C52. The network comprises a core layer (DAR-DEG intersections) and an extended layer (DAR-only). Circular nodes represent candidate TFs, and square nodes represent downstream target genes. Node size is proportional to the total degree of connectivity (in-degree + out-degree), reflecting regulatory importance. Edge thickness indicates the statistical confidence (E-value) of the Motif-TF match. Node colors correspond to distinct WGCNA modules (e.g., *CsTGA7* co-segregates with *CsDMR6* and *CsFTIP7*). **(E)** Expression heatmap of candidate TFs under NN and LN conditions. Key stress-responsive families (bZIP, AP2/ERF, WRKY) are highlighted, with Cs*TGA7* showing significant upregulation and *CsERF118* showing downregulation (*P* < 0.05). **(F)** Integrative visualization of chromatin accessibility (IGV tracks) and relative gene expression (bar charts) for four core target genes (Cs*DMR6*, *CsFTIP7*, *CsAHG1*, *CsSDR3b*). Red boxes highlight regulatory regions where chromatin accessibility decreased under low (N) Note the divergent transcriptional outcomes: *CsDMR6* and *CsSDR3b* were downregulated (concordant), whereas *CsFTIP7* and *CsAHG1* were upregulated (discordant), suggesting diverse chromatin-based regulatory mechanisms.

To decipher the chromatin accessibility heterogeneity underlying the differential N-stress tolerance between C52 (tolerant) and C13 (sensitive), we performed ATAC-seq on the second newly emerged leaves under normal (NN) and N-deficient (LN, 4 h) conditions. As shown in **Figures 4A**, a distinct enrichment of open chromatin signals was observed around the Transcription Start Sites (TSS) in both accessions regardless of N status. This distribution pattern, characteristic of high-quality ATAC-seq libraries, confirms the reliability of the dataset in capturing chromatin accessibility dynamics at key regulatory regions.

To quantify N-starvation-induced chromatin remodeling, we identified Differentially Accessible Regions (DARs; *P*-value < 0.05, |log_2_FC| > 0.5) and Differentially Expressed Genes (DEGs; *P*-value < 0.05, |log_2_FC| > 1). The UpSet plot (**Figure 4B**) illustrates the intersection between DARs and DEGs. While N deficiency triggered widespread changes in both chromatin accessibility and gene transcription, the overlap was not absolute. To further explore the coordination between chromatin accessibility changes and transcriptional output, we performed a Pearson correlation analysis on the union of significant features (**Supplementary Figures 1A, B**). The global correlation was relatively weak in both accessions (C52: R = 0.063; C13: R = 0.26), suggesting a multilayered regulatory landscape where chromatin remodeling acts as a prerequisite or priming event rather than a linear determinant of gene expression.

To dissect this complexity, we conducted a nine-quadrant analysis to classify genes based on their regulatory modes (**Supplementary Figures 1A, B**). In the sensitive accession C13, no genes were significantly enriched in the quadrants of interest. In contrast, C52 exhibited specific functional clusters. Notably, key stress-responsive genes, including *CsDMR6*, *CsSDR3*, *CsWEE1*, *CsDUF584*, and *CsUGT71C3*, were classified into the “Hypo-Down” quadrant (reduced accessibility coupled with downregulation), following a canonical positive regulatory mode. Interestingly, *CsFTIP7* and *CsAHG1* fell into the “Hypo-Up” quadrant (reduced accessibility coupled with upregulation), hinting at a distinct repressive mechanism. This classification provided a specific candidate list for identifying direct upstream drivers.

To identify these direct drivers of the transcriptional response, we focused on the intersection of DARs and DEGs, particularly in the tolerant accession C52. We extracted genomic sequences flanking the DAR peaks (± 1 kb) of these intersecting genes and performed motif enrichment analysis using three complementary algorithms: HOMER, MEME-ChIP, and PlantTFDB. By matching enriched motifs to known Transcription Factors (TFs) via TOMTOM and identifying the consensus across methods (**Figure 4C**), we established a high-confidence set of candidate regulators. **Supplementary Figure 2** highlights the most significantly enriched motifs within core DARs, predominantly belonging to stress-responsive families. Notably, the bZIP family appeared with the highest frequency (Motifs 6, 7, 8, 9, 10, 12, 13), suggesting a central regulatory role. Conserved motifs for WRKY (Motifs 4, 5), AP2/ERF (Motifs 2, 15), and C2C2-GATA (Motif 1, E-value = 6.6 × 10^-7^) were also significantly enriched.

Based on these high-confidence TF-motif pairs, we constructed a Transcriptional Regulatory Network (TRN) for the early N-starvation response in C52 (**Figure 4D**). The network features a hierarchical “multi-TF to multi-target” architecture, comprising a core layer (DAR-DEG intersecting genes, e.g., *DMR6*, *SDR3b*) and an extended layer (DAR-only genes). In this network, node size reflects the regulatory connectivity (total degree), and edge thickness indicates the statistical confidence of the TF-motif match. Integrating WGCNA data from the 30 h time point revealed that certain TF-target pairs (e.g., *TGA7* targeting *DMR6* and *FTIP7*) co-segregate into the same expression module, pointing to sustained regulatory modules.

To validate these predictions, we analyzed the expression profiles of candidate TFs (**Figure 4E**). Consistent with the motif enrichment, the heatmap revealed significant transcriptional modulation of bZIP, AP2/ERF, and WRKY family members. Specifically, *TGA7* (bZIP family) was significantly upregulated, whereas *ERF118* was significantly downregulated under low N conditions (*P* < 0.05). Integrative visualization using IGV tracks (**Figure 4F**) provided mechanistic insights into the regulation of four core target genes (*DMR6*, *FTIP7*, *AHG1*, *SDR3b*) in the nine-quadrant analysis. Low nitrogen treatment induced a marked reduction in chromatin accessibility at the promoter/regulatory regions of these genes, an effect more pronounced in C52. Interestingly, the transcriptional outcomes diverged: Consistent with their quadrant classification, *DMR6* and *SDR3b* (Hypo-Down) showed downregulation concomitant with reduced accessibility, following a canonical positive regulatory mode. In contrast, *FTIP7* and *AHG1* (Hypo-Up) were upregulated despite chromatin closing (**Figures 4F**, bar charts). This “discordant” pattern suggests a complex regulatory mechanism, potentially involving the displacement of transcriptional repressors or the competitive binding of activators/repressors during chromatin remodeling.

In summary, C52 orchestrates a rapid and precise low-nitrogen response by remodeling chromatin landscape to differentially regulate functionally distinct gene sets. The identification of key TFs, such as *TGA7*, and their distinct regulatory modes (concordant vs. discordant) highlights the sophistication of the chromatin accessibility-transcriptional machinery in the N-tolerant genotype.

### 3.4 *CsTGA7* orchestrates low-nitrogen tolerance by maintaining nitrate homeostasis and sustaining stress-responsive gene expression

**Figure 5:**
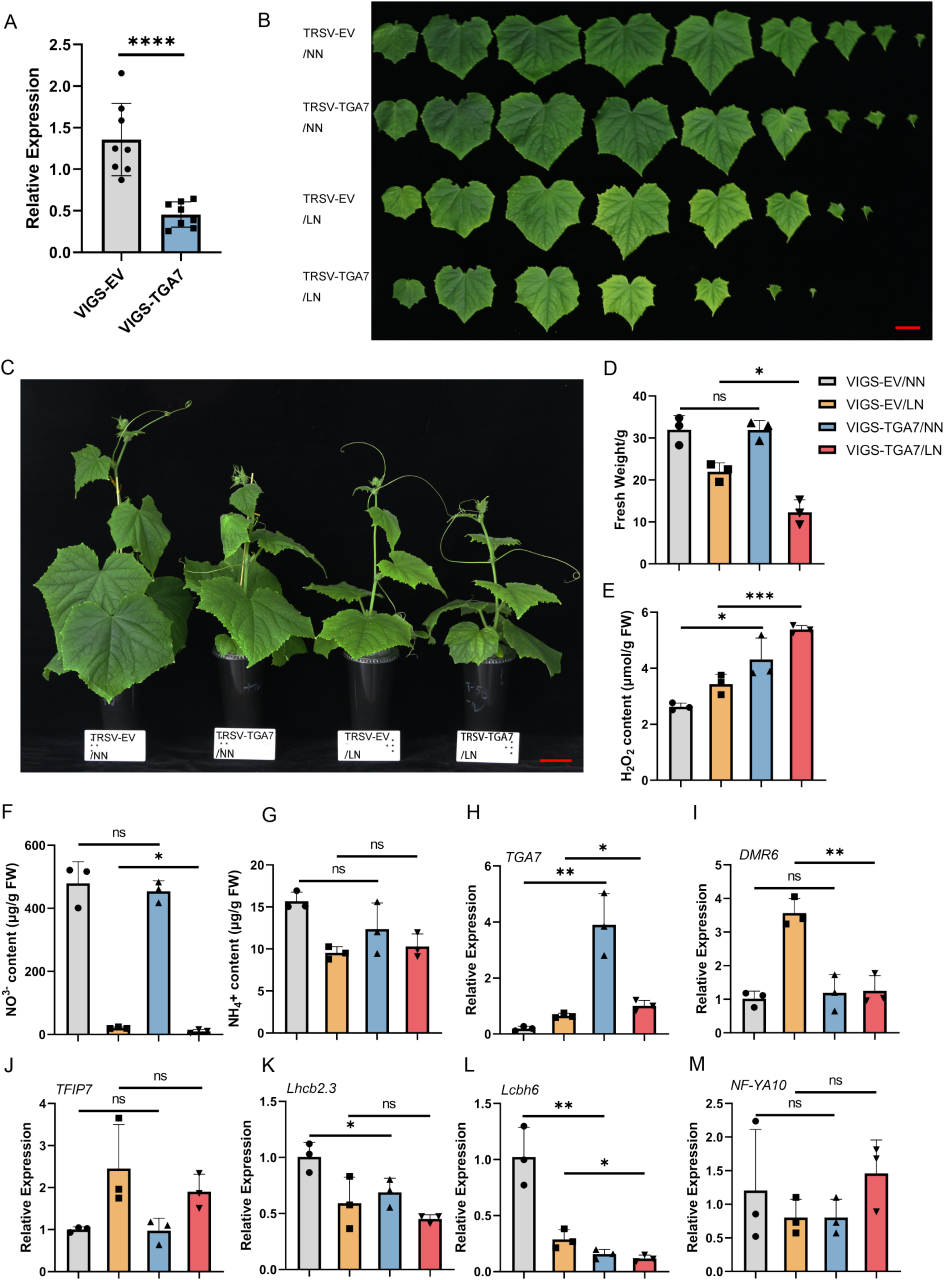
Functional validation of *CsTGA7* in regulating low-nitrogen tolerance and nitrate homeostasis using VIGS. **(A)** Verification of silencing efficiency. Relative expression levels of *CsTGA7* in empty vector control (TRSV-EV) and *CsTGA7*-silenced (TRSV-TGA7) plants were measured by qRT-PCR. **(B, C)** Phenotypic characterization. Representative images of TRSV-EV and TRSV-TGA7 plants grown under normal nitrogen (NN) or low nitrogen (LN) conditions for 7 days. Note the visible leaf yellowing and growth retardation in silenced plants under LN stress. **(D–G)** Physiological measurements and growth analysis. Quantification of shoot fresh weight **(D)**, H_2_O_2_ content **(E)**, nitrate (NO_3_^-^) content **(F)**, and ammonium (NH_4_^+^) content **(G)**. *CsTGA7* silencing resulted in biomass reduction, ROS accumulation, and impaired nitrate accumulation under LN stress. **(H–M)** Expression profiles of *CsTGA7* and its downstream targets. qRT-PCR analysis of *CsTGA7* **(H)**, *CsDMR6* **(I)**, *CsFTIP7* **(J)**, *CsLhcb2.3* **(K)**, *CsLhcb6* **(L)**, and *CsNF-YA10* **(M)** in gene-silenced and control plants under NN and LN conditions. Data are presented as means ± SD (n ≥ 3). Asterisks indicate statistically significant differences determined by Student’s t-test (*P < 0.05; **P < 0.01; ***P < 0.001; ****P < 0.0001). “ns” indicates no significant difference.

To elucidate the physiological role of *CsTGA7* in adaptation to nitrogen (N) deficiency, we generated *CsTGA7*-silenced plants using the Tobacco Ringspot Virus (TRSV)-based VIGS system. Following the appearance of photobleaching in the TRSV-PDS positive control (**Supplementary Figure 1C**), qRT-PCR analysis confirmed a significant reduction in *CsTGA7* transcript levels in TRSV-TGA7 plants compared to the empty vector (TRSV-EV) controls (**Figure 5A**). The plants were subsequently subjected to normal nitrogen (NN) or low nitrogen (LN) conditions for 7 days.

Unlike the early transcriptional changes observed at 4 h, long-term deficiency imposed a discernible growth constraint on *CsTGA7*-silenced plants.Under low N conditions, TRSV-TGA7 lines exhibited a sensitive phenotype characterized by visible leaf yellowing and biomass reduction (**Figures 5B–D**). Physiological dissection revealed that this growth defect was accompanied by a specific reduction in nitrate accumulation: while ammonium levels remained unaffected, nitrate content was significantly lower in silenced plants compared to controls (**Figures 5F, G**). Furthermore, the elevated accumulation of H_2_O_2_ (**Figure 5E**) served as a physiological indicator of aggravated stress, confirming that *CsTGA7*-silenced plants failed to maintain redox homeostasis under N deprivation.

To understand the molecular basis of these defects, we examined the distinct regulatory modules predicted by our multi-omics network. The expression of the core target *CsDMR6* (Van Damme et al., 2008), a putative 2-oxoglutarate Fe(II)-dependent oxygenase known to mediate salicylic acid (SA) homeostasis, exhibited a distinct temporal pattern.exhibited a distinct temporal pattern. Furthermore, qRT-PCR analysis confirmed that CsTGA7 remained suppressed in silenced plants after 7 days of N starvation (**Figure 5H**). While downregulated at 4 h (early shock phase), *CsDMR6* was significantly upregulated in control plants after 7 days of N starvation, likely reflecting an adaptive recovery mechanism. Strikingly, this adaptive upregulation was completely compromised in TRSV-TGA7 plants (**Figure 5I**). This places *CsTGA7* as a critical “sustainer” required to maintain high levels of defense/stress genes during prolonged starvation. Consistent with the severe chlorosis phenotype, silencing *CsTGA7* led to a broad repression of light-harvesting genes. *CsLhcb6* was suppressed under all conditions, and *CsLhcb2.3* failed to maintain basal expression levels (**Figures 5K, L**). This suggests that *CsTGA7* contributes to tolerance partially by protecting the photosynthetic machinery from N-deprivation induced senescence. Notably, not all predicted targets showed dependency on *CsTGA7* at this stage. The expression of *CsTFIP7* and *NF-YA10* remained unaltered in silenced plants (**Figures 5J, M**). This indicates that while these genes are co-expressed with *CsTGA7* (as shown in WGCNA), their regulation under long-term stress may involve redundant pathways or alternative transcription factors, highlighting the specificity of the TGA7-mediated regulatory branch.

## 4 Discussion

Nitrogen (N) is a key determinant of cucumber yield, yet the hierarchical regulatory networks governing N adaptation in horticultural crops remain largely uncharted compared to model species. While transcriptional reprogramming under N stress is well-documented, the upstream chromatin-level events that gate these responses are less understood. In this study, by integrating time-course RNA-seq and ATAC-seq, we deciphered the regulatory logic distinguishing a tolerant cucumber accession (C52) from a sensitive one (C13). Our data revealed that N tolerance is not merely about specific gene induction but relies on a systemic strategy: C52 employs an active “resource recycling” mechanism underpinned by the ubiquitin-proteasome system (UPS), whereas C13 succumbs to a passive “ chromatin-mediated dormancy”.

Our comparative WGCNA analysis highlighted fundamentally different survival strategies between the two accessions. The tolerant accession C52 was characterized by the significant enrichment of the UPS pathway in the “RED” module. In plants, the targeted degradation of proteins via ubiquitination is a critical mechanism for remobilizing amino acids from senescing tissues to support young leaf growth under N starvation (Liu et al., 2017). The activation of proteasome core components (e.g., *PSMA4*, *RBX1*) suggests that C52 actively “recycles” non-essential or damaged proteins to fuel metabolic flexibility. In stark contrast, the sensitive accession C13 exhibited global chromatin tightening and a broad downregulation of translational machinery as shown in Result 3. While chromatin compaction can serve as a defensive mechanism to protect genomic integrity from stress-induced damage, it imposes a “dormant” state that restricts transcriptional plasticity (Dar et al., 2022; Lloyd and Lister, 2022; Liu et al., 2025). This “state of restricted chromatin accessibility lockdown” likely prevents the rapid activation of stress-responsive genes, explaining the transcriptional inertia and severe chlorosis observed in C13. These findings underscore that metabolic plasticity, driven by efficient recycling rather than passive conservation, is the key determinant of LN tolerance in cucumber.

The selection of 4 h and 30 h post-treatment time points was strategically designed to capture the distinct “regulatory priming” and “functional adaptation” phases of the cucumber low nitrogen (LN) response. Nitrogen deficiency is a progressive physiological stress; however, our primary objective was to identify the “first responder” transcription factors that command the early signaling cascade. According to previous transcriptomic profiling in cucumber seedlings, the most intense wave of gene regulation typically occurs between 3 and 6 h post-treatment (Zhao et al., 2015). By sampling at 4 h, we targeted the “golden window” for ATAC-seq to capture early chromatin remodeling events that precede or coincide with this primary transcriptional peak. The subsequent 30 h time point allowed us to observe the stabilized steady-state expression of downstream metabolic effectors, such as nutrient transporters and recycling enzymes, thereby bridging the gap between initial chromatin-level signaling and long-term physiological adaptation. This temporal integration provides a high-resolution snapshot of how early chromatin accessibility changes dictate the trajectory of sustained LN tolerance.

The integration of ATAC−seq provided mechanistic insights that RNA−seq alone could not reveal. A subset of key stress−responsive genes displayed a discordant regulatory pattern, in which transcript levels increased despite reduced chromatin accessibility (**Figure 4F**). Representative examples include the ABA−related regulator *CsAHG1 CsAHG1* (Krüger et al., 2025) and the intracellular transport-related gene *CsFTIP7* (Jiang et al., 2021). Rather than contradicting established principles, this pattern reflects the inherent complexity of chromatin–transcription relationships. Chromatin accessibility is a permissive but not deterministic layer of regulation, and several mechanisms can uncouple accessibility from transcriptional output. One plausible explanation is stress−induced displacement of transcriptional repressors: localized chromatin condensation may destabilize repressor binding, thereby relieving transcriptional inhibition even when accessibility decreases (Zhu, 2016; Wang et al., 2022). Alternatively, epigenetic modifications, such as histone acetylation or H3K4/H3K27 dynamics, may override accessibility changes to sustain transcription under stress. These observations highlight that cucumber employs context−dependent and multilayered chromatin regulation during LN adaptation. In the tolerant accession C52, such fine−tuned chromatin remodeling likely contributes to the coordinated activation of pathways related to hormonal crosstalk and macromolecule transport, enabling a more precise and resilient transcriptional response to N deprivation.

TGA (TGACG-motif binding) transcription factors, belonging to the basic leucine zipper (bZIP) family, serve as pivotal regulatory hubs integrating multiple hormonal signals and environmental stress responses. Over the past two decades, extensive research has demonstrated that TGA members interact with diverse regulatory proteins—including NPR1, GRX480, ERF72, and SCL14-to modulate signaling pathways mediated by salicylic acid (SA), jasmonic acid (JA), ethylene (ETH), and cytokinins (CK). In the context of biotic defense, TGA1–TGA7 are well characterized for their interaction with NPR1, the master regulator of the SA pathway, to activate systemic acquired resistance through the induction of PR genes. Beyond defense, TGAs are essential for maintaining cellular redox homeostasis by regulating antioxidant enzymes such as catalases (CAT) and glutathione S-transferases (GST) (Lu et al., 2024). For instance, overexpression of *ScTGA1* in sugarcane reduces H_2_O_2_ accumulation and enhances pathogen tolerance (Zhao et al., 2023). More recently, the role of TGAs in nutrient sensing has gained prominence. In Arabidopsis, TGA1 and TGA4 directly bind to the promoters of nitrate transporters *NRT2.1* and *NRT2.2*. Overexpression of *AtTGA4* promotes root development under low-N conditions and induces nitrate reductase genes *NIA1* and *NIA2*, synergistically improving N uptake and assimilation efficiency (Alvarez et al., 2014).

Based on the integrated analysis of ATAC-seq and RNA-seq, we identified *CsTGA7* (and *CsERF118*) as a core regulatory hub of the LN response. To ensure high-confidence selection, we utilized an intersection of three independent motif-discovery algorithms (HOMER, MEME-ChIP, and PlantTFDB) within DARs, complemented by concordant expression dynamics and reproducible VIGS phenotypes. Comparative analyses further support the functional significance of *CsTGA7*. Pairwise global alignment between CsTGA7 (372 aa) and AtTGA7 (368 aa) revealed 54.8% amino-acid identity and 69.9% similarity, with a highly conserved bZIP (BRLZ) DNA-binding domain and DOG1-like features (**Supplementary Figures 3A, B**). This high degree of structural conservation, particularly in the DNA-binding region, provides a molecular basis for the conserved regulatory motifs we identified. Phylogenetic analysis places CsTGA7 within a well-supported cucurbit clade (**Supplementary Figure 3C**). While DNA-binding specificity appears conserved, the functional divergence observed between species may arise from variations in species-specific regulatory contexts.

Our findings position CsTGA7 as a potential systemic integrator of N status, mirroring the recently discovered role of its ortholog, AtTGA7, as a shoot-to-root mobile protein (Ye et al., 2025). In Arabidopsis, *TGA7* is induced in the shoot vasculature under N-limitation and translocated to the roots to orchestrate a dual-regulatory program: activating light-harvesting genes (e.g., Lhcb2.3 and Lhcb6) in the shoot to safeguard photosynthesis, and enhancing N-acquisition in the root by activating *NRT2.1* or modulating *NRT2.4/2.5* through the NF-YA10 cascade. In our assays, VIGS-mediated silencing of CsTGA7 compromised both leaf photosynthetic gene expression and nitrate accumulation, consistent with this conserved framework. We hypothesize that CsTGA7 may coordinate systemic resource allocation in cucumber, potentially through a similar transcriptional cascade involving light-harvesting factors and nitrate transporters. A key mechanistic hypothesis from our study is the potential regulation of *CsDMR6* by CsTGA7. CsDMR6 encodes an SA 5-hydroxylase that degrades SA to prevent immune overactivation. We propose that the CsTGA7-dependent reactivation of *CsDMR6* under prolonged starvation acts as a “metabolic brake” to prevent SA hyper-accumulation and potentially mitigate subsequent oxidative damage. The failure of *CsTGA7*-silenced plants to maintain *CsDMR6* expression coincides with significant H_2_O_2_ accumulation, suggesting that this CsTGA7-*CsDMR6* regulatory module is likely critical for redox balance (Van Damme et al., 2008; Thomazella et al., 2021; Giacomelli et al., 2023). This role contrasts with the AtFOX1-TGA7 module in Arabidopsis, which promotes senescence by increasing ROS (Zhao et al., 2025). Our observation that silencing CsTGA7 leads to H_2_O_2_ elevation under both NN and LN conditions suggests that in cucumber, CsTGA7 may act as a fundamental guardian of basal redox homeostasis.

While our integrative multi-omics and VIGS data strongly nominate CsTGA7 as a central regulator, we acknowledge that these results do not yet constitute direct *in vivo* binding proof or a definitive causal hierarchy. To fully address the mechanistic depth requested, our future work will focus on: (1) Mapping the direct *in vivo* cistrome of CsTGA7 using DAP-seq or ChIP-seq to confirm its binding to CsDMR6 and CsLhcb promoters; (2) Generating stable CRISPR/Cas9 knockout and overexpression lines to characterize the long-term impact on nitrate uptake and yield; and (3) Quantifying temporal changes in SA levels and NR activity in homozygous transgenic lines to refine our “metabolic brake” model. These efforts will further unravel the sophisticated wiring of this conserved N-signaling hub and its hierarchical relationship with primary N-sensors in cucurbits.

## 5 Conclustion

This study provides a comprehensive multi-omics roadmap of the chromatin-based and transcriptional mechanisms governing low-nitrogen (LN) tolerance in cucumber. We revealed that LN adaptation relies on a strategic shift from passive “ chromatin-mediated dormancy”—characterized by chromatin tightening in sensitive genotypes—to an active “resource recycling” strategy underpinned by the ubiquitin-proteasome system in tolerant genotypes. Crucially, by integrating chromatin accessibility dynamics with transcriptomic data, we identified and functionally validated the bZIP transcription factor *CsTGA7* as a core regulator of this adaptive response. Our results demonstrate that *CsTGA7* is indispensable for coordinating nitrate homeostasis, maintaining redox balance, and sustaining the expression of photosynthesis-related genes under prolonged N starvation. These findings not only advance our understanding of the hierarchical gene regulatory networks in horticultural crops but also establish *CsTGA7* as a promising genetic target for breeding low-nitrogen tolerant cultivars to promote sustainable vegetable production.

## Data Availability

The datasets presented in this study can be found in the NCBI BioProject repository, accession number PRJNA1414160 (https://www.ncbi.nlm.nih.gov/bioproject/PRJNA1414160).

## Supplementary material

Supplementary Figure 1 Global correlation analysis of chromatin accessibility and transcriptional changes in C52 and C13 under early low−nitrogen stress. (A, B) Nine−quadrant scatter plots illustrating the relationship between differential chromatin accessibility (ATAC−seq log2FC, *y−axis*) and differential gene expression (RNA−seq log2FC, *x−axis*) in the (A) tolerant accession C52 and (B) sensitive accession C13. The analysis was performed on the union of genes showing significant changes (*P* < 0.05) in either dataset. Vertical and horizontal dashed lines indicate the fold−change thresholds: |log2FC| > 1 for RNA−seq and |log2FC| > 0.5 for ATAC−seq. Genes falling into specific regulatory quadrants are colored and labeled as follows: Hyper−Up: Increased chromatin accessibility coupled with upregulated expression; Hypo−Down: Decreased accessibility coupled with downregulated expression (canonical positive correlation); Hypo−Up: Decreased accessibility but upregulated expression (discordant/repressive release); Hyper−Down: Increased accessibility but downregulated expression; Grey dots represent genes that did not meet both fold−change thresholds. Key stress−responsive genes in C52-including *CsDMR6*, *CsSDR3*, *CsWEE1*, *CsDUF584*, and *CsUGT71C3* (Hypo−Down), as well as *CsFTIP7* and *CsAHG1* (Hypo−Up)—are highlighted in panel (A). Pearson correlation coefficients (*R*) and *P*−values are displayed within each plot. Notably, C52 exhibits distinct clustering of functional genes in specific quadrants, whereas C13 shows no significant quadrant enrichment. (C) Positive control for VIGS efficiency. Photobleaching phenotype of TRSV-PDS plants demonstrating successful virus-induced gene silencing.

Supplementary Figure 2 Significantly enriched transcription factor binding motifs within core differentially accessible regions (DARs).This table summarizes the top enriched motifs identified in the core DARs, defined as regions consistently responsive to low−nitrogen stress in both the LN−tolerant and LN−sensitive accessions. Columns include the consensus motif sequence logo, statistical significance (E−value), the reference database used for annotation (e.g., JASPAR Core Plants, Arabidopsis, or Cucumis sativus), and the predicted transcription factor (TF) family. Motifs are ranked according to enrichment significance.

Supplementary Figure 3 Sequence conservation and phylogenetic analysis of CsTGA7. **(A)** Pairwise protein sequence alignment of Arabidopsis AtTGA7(AT1G77920.1) and cucumber CsTGA7(CsaV3_2G033550.1). Global alignment was performed using EMBOSS Needle (BLOSUM62 matrix), showing aligned sequences, conserved residues, and similarities. The alignment reveals a high degree of conservation with 54.8% identity (215/392 residues), 69.9% similarity (274/392 residues), and 11.2% gaps (44/392 residues). **(B)** Domain architecture of AtTGA7 and CsTGA7. Schematic representation shows the conserved basic region leucine zipper (BRLZ/bZIP) DNA-binding domain and the DOG1-like region. Domain boundaries were annotated using InterProScan and Pfam. The scale bar represents 100 amino acids (aa). **(C)** Phylogenetic relationship of CsTGA7 and representative TGA7 orthologs. The Maximum Likelihood (ML) tree was constructed from full-length protein sequences. Bootstrap support values are indicated at the nodes. CsTGA7 clusters within a well-supported cucurbit-specific clade, including orthologs from Cucumis melo (melon), Citrullus lanatus (watermelon), Lagenaria siceraria (bottle gourd), and Cucurbita maxima (squash).

Supplementary Figure 4 Reproducibility and quality assessment of RNA-seq and ATAC-seq datasets. **(A)** Principal Component Analysis (PCA) of RNA-seq samples. The PCA plot illustrates the clustering of all 24 independent biological replicates based on their global gene expression profiles. PC1 and PC2 account for 46.74% and 25.07% of the total variance, respectively. Samples are colored according to their respective experimental groups (genotype * treatment * time point). **(B)** Pearson correlation heatmap of RNA-seq samples. Sample-to-sample correlation analysis of the 24 transcriptome profiles. The color intensity corresponds to the R^2^ value. **(C)** Pearson correlation heatmap of ATAC-seq samples. Correlation analysis for the 8 ATAC-seq samples (2 genotypes * 2 treatments * 2 biological replicates) based on normalized read counts across all identified peaks. The numbers within the cells represent the Pearson correlation coefficient (R), confirming robust data consistency between replicates.

Supplementary Table 1 List of primers used in this study. This file includes primer sequences, and their specific applications for qRT-PCR and VIGS cloning.

Supplementary File 1 Comprehensive datasets of transcriptional and chromatin accessibility changes. This compressed archive (ZIP format) contains three separate files: (1) List of differentially expressed genes (DEGs) identified by RNA-seq at 4 h under low-nitrogen stress; (2) List of DEGs identified by RNA-seq at 30 h; and (3) List of differentially accessible regions (DARs) identified by ATAC-seq at 4 h.
