# Supplementary figures and images for: Integration of ATAC-seq and RNA-seq unveils transcription factors involved in low-nitrogen tolerance in cucumber
Source: Front Plant Sci. 2026 Mar 26;17:1798277. doi: 10.3389/fpls.2026.1798277 (PMC13062244; doi:10.3389/fpls.2026.1798277)

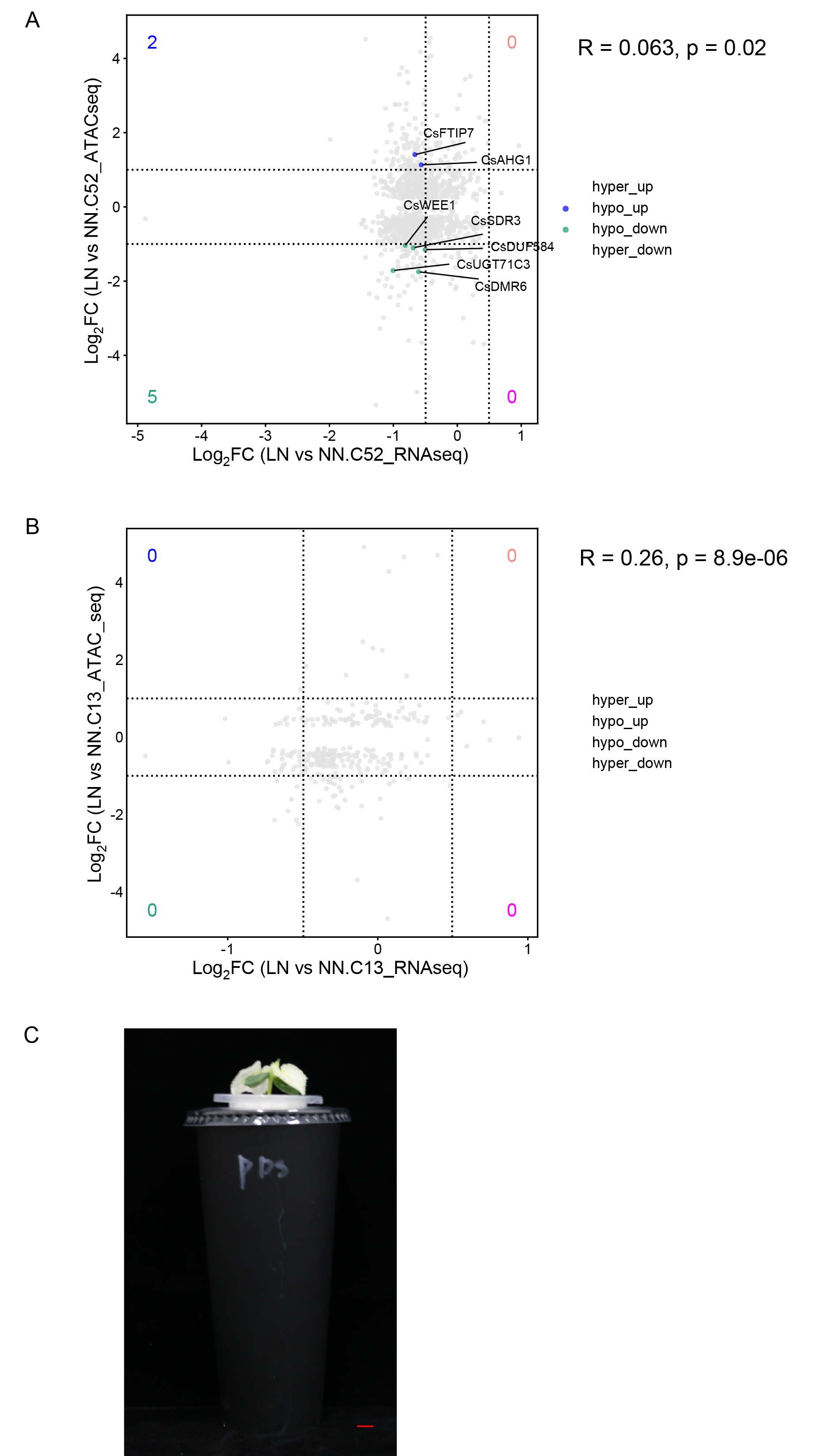

Supplement: Supplementary Figure 1 — Global correlation analysis of chromatin accessibility and transcriptional changes in C52 and C13 under early low−nitrogen stress. (A, B) Nine−quadrant scatter plots illustrating the relationship between differential chromatin accessibility (ATAC−seq log2FC, y−axis) and differential gene expression (RNA−seq log2FC, x−axis) in the (A) tolerant accession C52 and (B) sensitive accession C13. The analysis was performed on the union of genes showing significant changes (P < 0.05) in either dataset. Vertical and horizontal dashed lines indicate the fold−change thresholds: |log2FC| > 1 for RNA−seq and |log2FC| > 0.5 for ATAC−seq. Genes falling into specific regulatory quadrants are colored and labeled as follows: Hyper−Up: Increased chromatin accessibility coupled with upregulated expression; Hypo−Down: Decreased accessibility coupled with downregulated expression (canonical positive correlation); Hypo−Up: Decreased accessibility but upregulated expression (discordant/repressive release); Hyper−Down: Increased accessibility but downregulated expression; Grey dots represent genes that did not meet both fold−change thresholds. Key stress−responsive genes in C52-including CsDMR6, CsSDR3, CsWEE1, CsDUF584, and CsUGT71C3 (Hypo−Down), as well as CsFTIP7 and CsAHG1 (Hypo−Up)—are highlighted in panel (A). Pearson correlation coefficients (R) and P−values are displayed within each plot. Notably, C52 exhibits distinct clustering of functional genes in specific quadrants, whereas C13 shows no significant quadrant enrichment. (C) Positive control for VIGS efficiency. Photobleaching phenotype of TRSV-PDS plants demonstrating successful virus-induced gene silencing. [file Image1.tif]

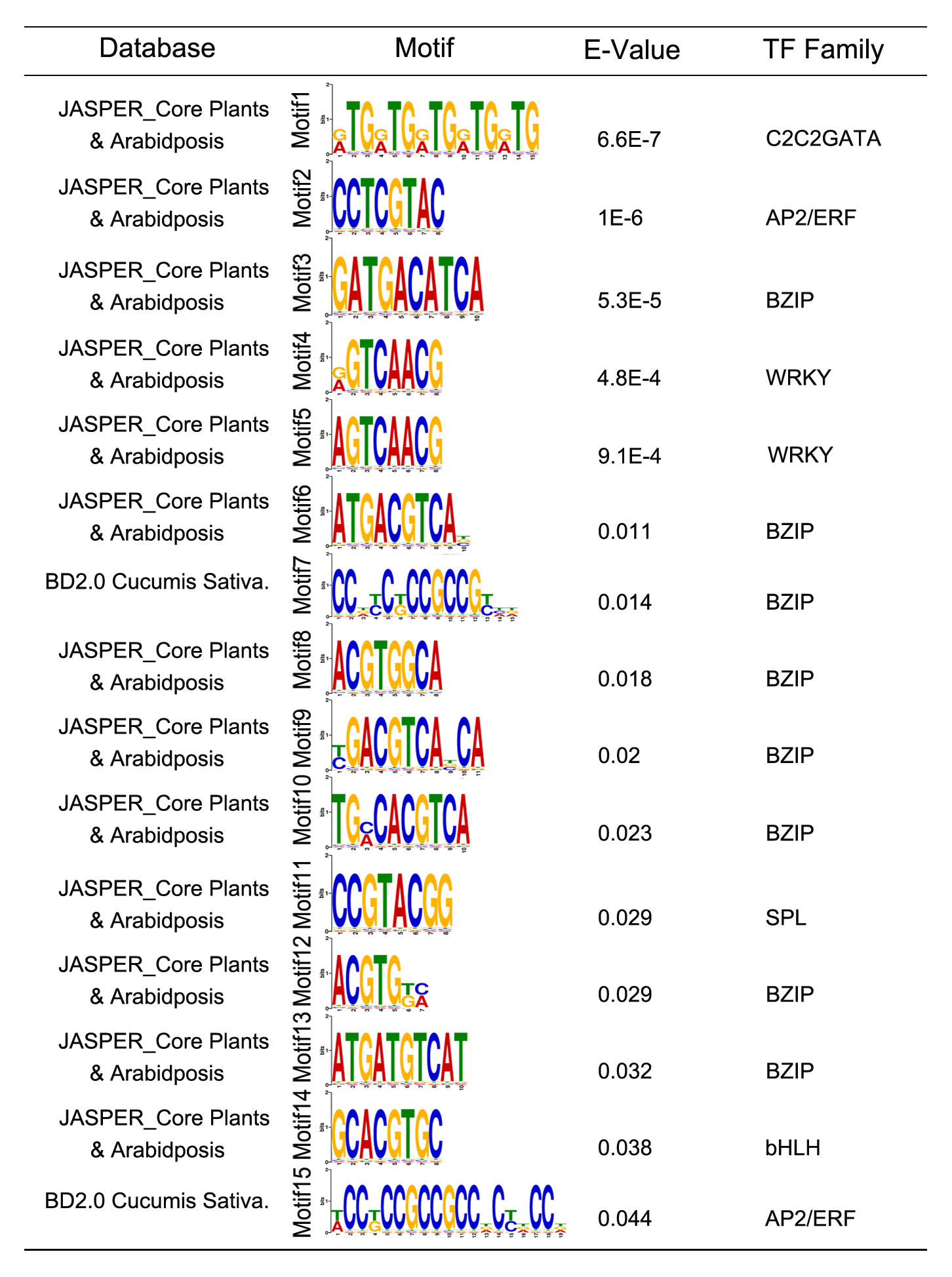

Supplement: Supplementary Figure 2 — Significantly enriched transcription factor binding motifs within core differentially accessible regions (DARs).This table summarizes the top enriched motifs identified in the core DARs, defined as regions consistently responsive to low−nitrogen stress in both the LN−tolerant and LN−sensitive accessions. Columns include the consensus motif sequence logo, statistical significance (E−value), the reference database used for annotation (e.g., JASPAR Core Plants, Arabidopsis, or Cucumis sativus), and the predicted transcription factor (TF) family. Motifs are ranked according to enrichment significance. [file Image2.tif]

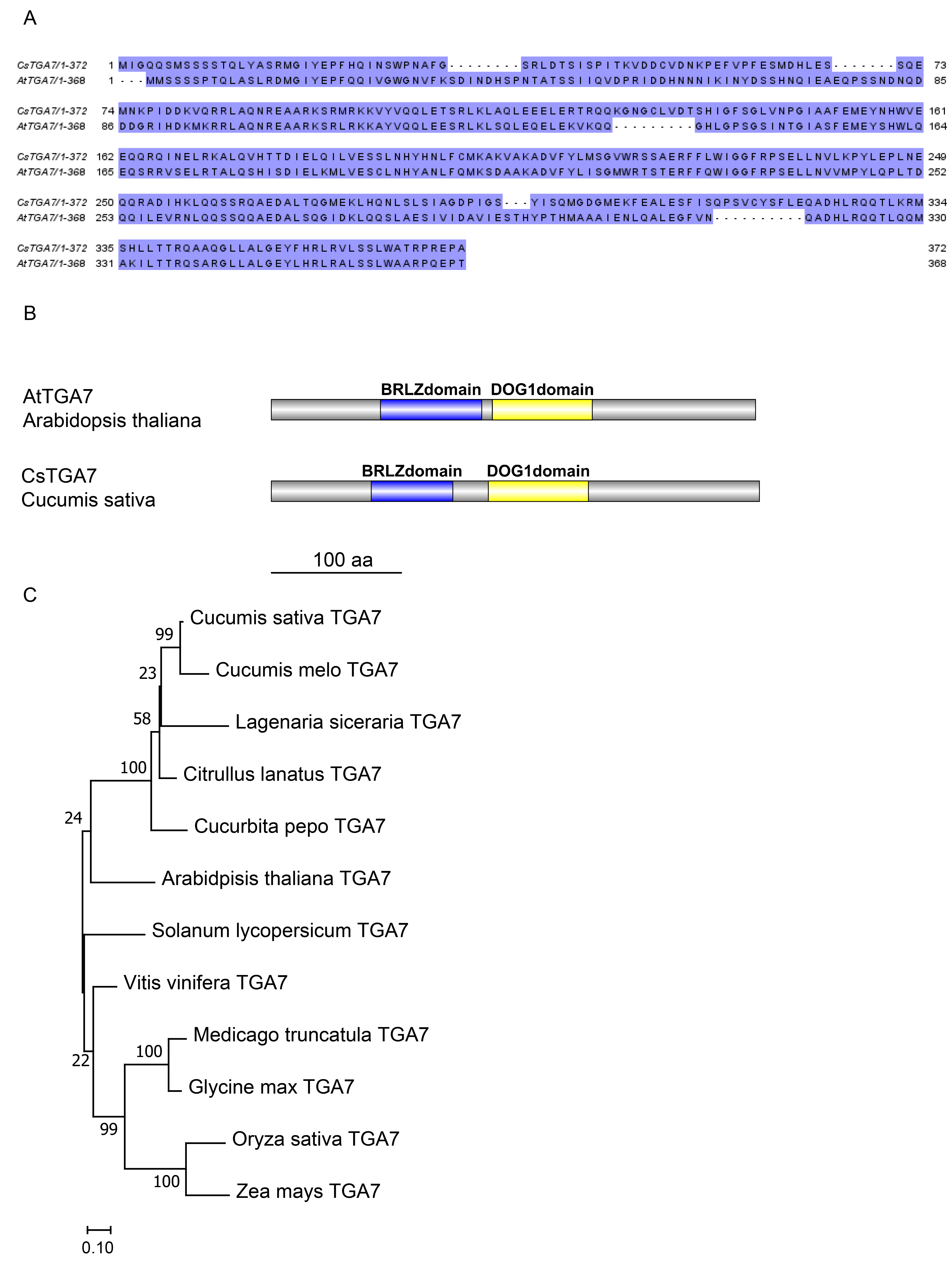

Supplement: Supplementary Figure 3 — Sequence conservation and phylogenetic analysis of CsTGA7. (A) Pairwise protein sequence alignment of Arabidopsis AtTGA7(AT1G77920.1) and cucumber CsTGA7(CsaV3_2G033550.1). Global alignment was performed using EMBOSS Needle (BLOSUM62 matrix), showing aligned sequences, conserved residues, and similarities. The alignment reveals a high degree of conservation with 54.8% identity (215/392 residues), 69.9% similarity (274/392 residues), and 11.2% gaps (44/392 residues). (B) Domain architecture of AtTGA7 and CsTGA7. Schematic representation shows the conserved basic region leucine zipper (BRLZ/bZIP) DNA-binding domain and the DOG1-like region. Domain boundaries were annotated using InterProScan and Pfam. The scale bar represents 100 amino acids (aa). (C) Phylogenetic relationship of CsTGA7 and representative TGA7 orthologs. The Maximum Likelihood (ML) tree was constructed from full-length protein sequences. Bootstrap support values are indicated at the nodes. CsTGA7 clusters within a well-supported cucurbit-specific clade, including orthologs from Cucumis melo (melon), Citrullus lanatus (watermelon), Lagenaria siceraria (bottle gourd), and Cucurbita maxima (squash). [file Image3.tif]

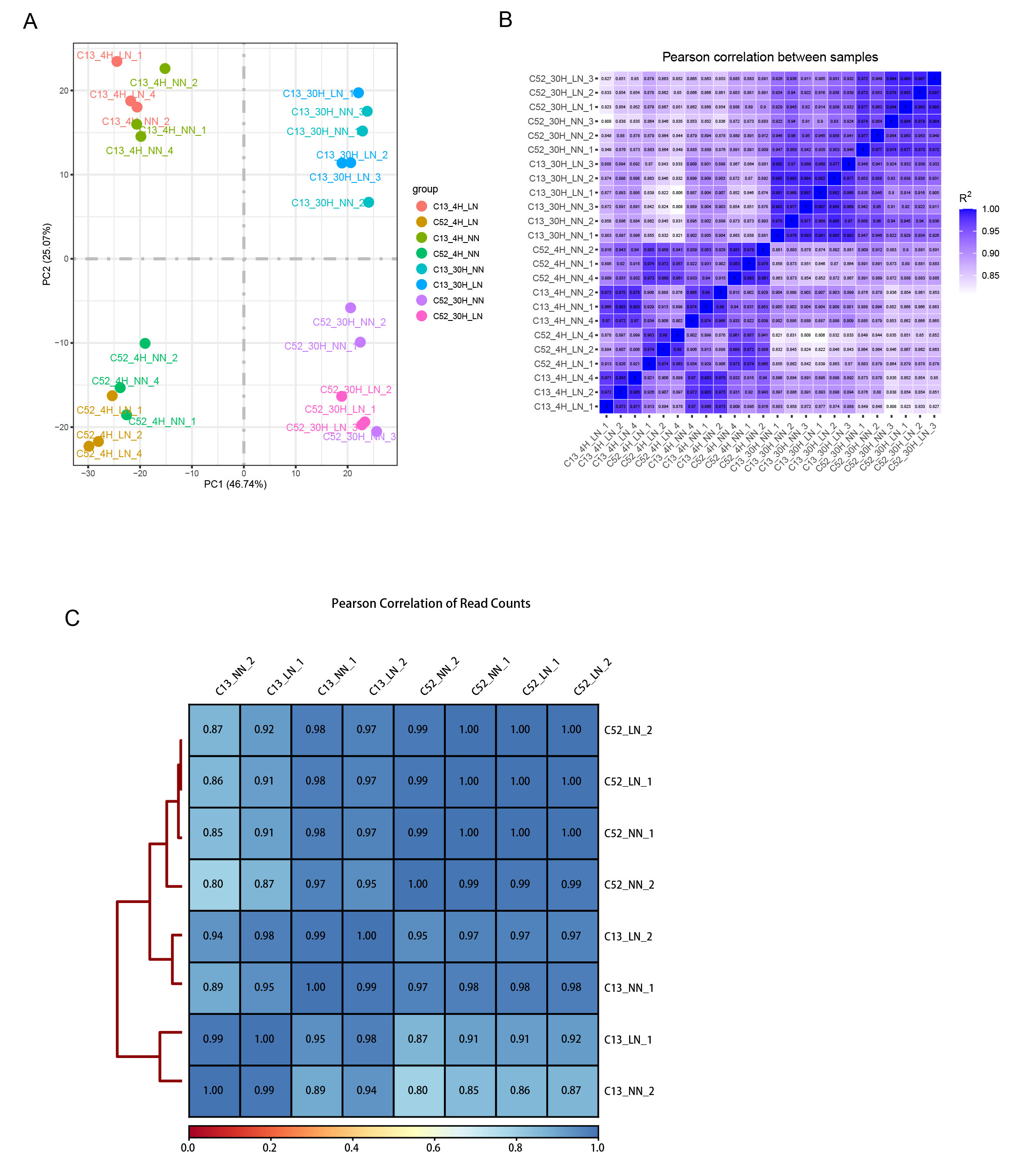

Supplement: Supplementary Figure 4 — Reproducibility and quality assessment of RNA-seq and ATAC-seq datasets. (A) Principal Component Analysis (PCA) of RNA-seq samples. The PCA plot illustrates the clustering of all 24 independent biological replicates based on their global gene expression profiles. PC1 and PC2 account for 46.74% and 25.07% of the total variance, respectively. Samples are colored according to their respective experimental groups (genotype * treatment * time point). (B) Pearson correlation heatmap of RNA-seq samples. Sample-to-sample correlation analysis of the 24 transcriptome profiles. The color intensity corresponds to the R2 value. (C) Pearson correlation heatmap of ATAC-seq samples. Correlation analysis for the 8 ATAC-seq samples (2 genotypes * 2 treatments * 2 biological replicates) based on normalized read counts across all identified peaks. The numbers within the cells represent the Pearson correlation coefficient (R), confirming robust data consistency between replicates. [file Image4.tif]
